# Prospective Evaluation of a Standardized Screening for Atrial Fibrillation after Ablation of Cavotricuspid Isthmus Dependent Atrial Flutter

**DOI:** 10.3390/jcm10194453

**Published:** 2021-09-28

**Authors:** Philipp Krisai, Laurent Roten, Ivan Zeljkovic, Nikola Pavlovic, Peter Ammann, Tobias Reichlin, Eric Auf der Maur, Olivia Streicher, Sven Knecht, Michael Kühne, Stefan Osswald, Jan Novak, Christian Sticherling

**Affiliations:** 1 Cardiovascular Research Institute Basel, Department of Cardiology, University Hospital Basel, 4031 Basel, Switzerland; philipp.krisai@usb.ch (P.K.); olivialea.streicher@usb.ch (O.S.); sven.knecht@usb.ch (S.K.); michael.kuehne@usb.ch (M.K.); stefan.osswald@usb.ch (S.O.); 2 Electrophysiology and Ablation Unit, Centre Hospitalier Universitaire de Bordeaux, 33600 Bordeaux-Pessac, France; 3 Department of Cardiology, Inselspital, Bern University Hospital, 3010 Bern, Switzerland; laurent.roten@insel.ch (L.R.); tobias.reichlin@insel.ch (T.R.); eric.aufdermaur@students.unibe.ch (E.A.d.M.); 4 Department of Cardiology, University Hospital Sestre Milosrdnice, 10000 Zagreb, Croatia; ivanzeljkov@gmail.com (I.Z.); nikolap12@yahoo.com (N.P.); 5 Department of Cardiology, Cantonal Hospital St. Gallen, 9007 St. Gallen, Switzerland; peter.ammann@kssg.ch; 6 Department of Cardiology, University Hospital Basel, 4031 Basel, Switzerland; 7 Herz-und Nierenzentrum Aare, 4500 Solothurn, Switzerland; jan.novak@hnz-aare.ch

**Keywords:** atrial fibrillation, sinus rhythm, electrocardiogram, cardiovascular outcomes, screening

## Abstract

Aims: We aimed to prospectively investigate the effectiveness of a standardized follow-up for AF-detection after common atrial flutter (cAFL) ablation. Methods: A total of 309 patients after cAFL ablation without known AF, from 5 centers, and at least one completed, standardized follow-up at 3, 6 and 12 months, including a 24 h Holter-electrocardiogram (ECG), were included. The primary outcome was incident atrial fibrillation (AF), or atrial tachycardia (AT). Predictors were investigated by Cox proportional-hazards models. Results: The mean age was 67.9 years; 15.2% were female and the mean CHA_2_DS_2_-VASc (Congestive heart failure, Hypertension, Age, Diabetes, Stroke, Vascular disease, Sex category) score was 2.4 points. The great majority of patients (90.3%) were anticoagulated. Over a mean follow-up of 12.2 months with a standardized approach, AF/AT was detected in 73 patients, corresponding to 11.7% at 3 months, 18.4% at 6 months and 28.2% at 12 months of follow-up. AF was found in 64 patients, AT in 9 and both in 2 patients. Occurrence of AF was recorded in 40 (60.6%) patients by Holter-ECG and in the remaining 26 (39.4%) by clinical follow-up only. There was no difference in male versus female (*p* = 0.08), or in younger versus older patients (*p* = 0.96) for AF/AT detection. Only coronary artery disease (hazard ratio [95% confidence intervals] 1.03 [1.01–1.05], *p* = 0.01) was associated with the primary outcome. Conclusions: AF or AT was detected in a large proportion of cAFL patients after cavotricuspid-isthmus (CTI) ablation, using a standardized follow-up over 1 year. This standardized screening can be easily implemented with high patient acceptance. The high proportion of post-ablation AF needs to be taken into consideration when deciding on long-term oral anticoagulation.

## 1. Introduction

Atrial fibrillation (AF) and cavotricuspid-isthmus (CTI) dependent, common atrial flutter (cAFL) frequently coexist [1,2]. Both arrhythmias share not only risk factors, but also direct arrhythmia mechanisms [3]. In the majority of cAFL patients, AF or supraventricular ectopy seems to be necessary to cause functional blockage between the vena cava, in order to enable re-entrant cAFL [4,5]. This close relationship between cAFL and AF has important clinical implications for patients presenting with lone cAFL, most importantly regarding the decision on long-term anticoagulation therapy. Currently, clear guideline recommendations are lacking for the preferred AF screening intensity or duration in cAFL patients after CTI catheter ablation [6]. Thus, clinicians are left in uncertainty about how to follow up with these patients in an efficient way.

We previously reported an AF incidence rate of 30%, with a follow-up of approximately two years in cAFL patients after CTI ablation in a retrospective analysis, with less stringent rhythm monitoring in one of the largest populations so far [2]. Based on these results, we set up a prospective, multi-center cohort study, to investigate the effectiveness and clinical utility of a standardized follow-up for AF screening after cAFL ablation. We also aimed to study independent predictors for AF occurrence, in order to be able to allocate screening resources to those patients who might benefit the most.

## 2. Materials and Methods

### 2.1. Patient Population

**Figure 1:**
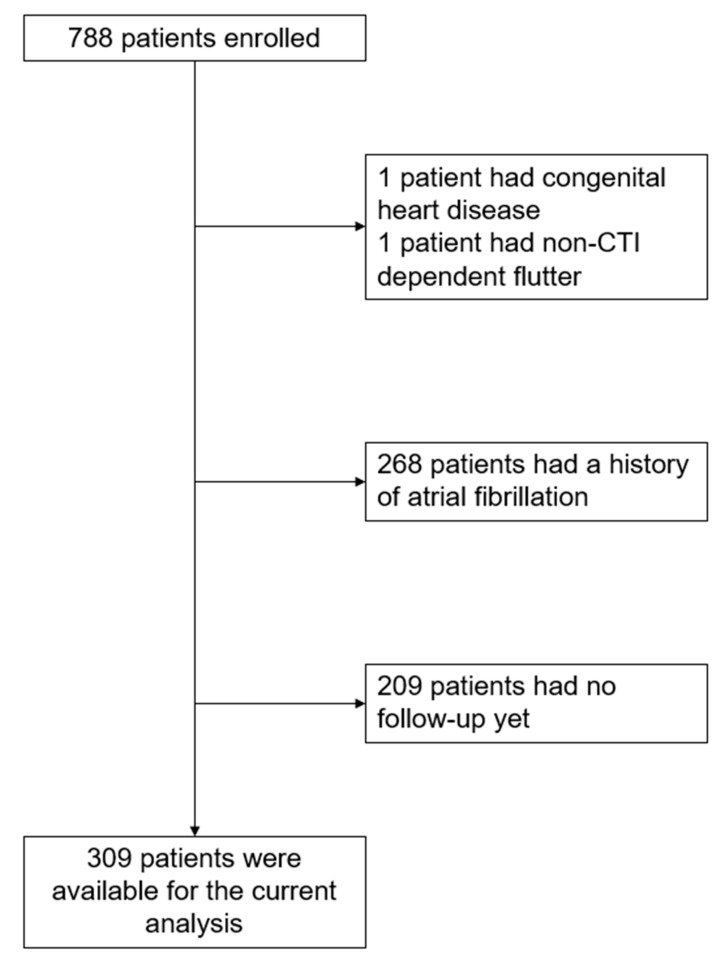
Study flow. CTI: cavotricuspid-isthmus.

Patients of the ongoing, prospective, multicenter BEAT-Flutter registry, with at least one follow-up visit, were included. In this registry, all patients undergoing CTI dependent cAFL ablation at five centers (four in Switzerland, one in Croatia) were included, from January 2017 to March 2021. Exclusion criteria were: patient refusal to participate, non-CTI-dependent AFL, prior heart surgery, and congenital heart disease. For the current exploratory analyses within the registry, we extracted the data in July 2021, and excluded all patients with a history of AF. The study flow is shown in Figure 1. Data acquisition and analysis were performed in compliance with protocols approved by the Ethical Committee Nordwest-und-Zentralschweiz (ethical approval number 2016-01865). Written informed consent was obtained from all participants prior to the study. The study was carried out according to the principles of the Declaration of Helsinki. The data supporting this study are available from the corresponding author upon reasonable request.

### 2.2. Assessment of Study Variables

Trained study personnel, using standardized questionnaires, obtained information on patient characteristics including demographics, medical history, and risk factors, during a clinical study visit on enrolment. We used the European Heart Rhythm Association (EHRA) score for symptom assessment. The EHRA score ranges from I to IV, with I indicating no symptoms, II indicating mild symptoms, III indicating severe symptoms, and IV indicating disabling symptoms [6]. We also assessed individual symptoms, including palpitations, angina pectoris, dizziness or syncope, and dyspnea. After catheter ablation for cAFL, patients were followed-up at three, six and twelve months at clinical outpatient visits, including 12-lead ECGs and 24 h Holter-ECGs at each visit. AF or AT had to be present for at least 30 s to be considered relevant. Follow-up visits were recommended to take place ±1 month for the 3- and 6-month follow-up visits and ±3 months for the 12-month follow-up visit. Patients were additionally advised to obtain ECG documentation, in case of arrhythmia symptoms at unscheduled clinical follow-ups.

### 2.3. Ablation Procedure

A linear lesion from the tricuspid annulus to the inferior vena cava was performed by radiofrequency ablation at 30–40 Watts, with either a non-irrigated 8 mm, or irrigated 3.5 mm tip catheter, under fluoroscopic and electrocardiogram guidance. The procedural endpoint was a bi-directional conduction block across the CTI [7]. Oral anticoagulation was not interrupted for the procedure.

### 2.4. Study Outcomes

The primary outcome was incident AF, or atrial tachycardia (AT), detected in a 12-lead ECG or 24 h Holter-ECG after catheter ablation of cAFL. AF or AT had to be confirmed by a cardiologist and present for at least 30 s. Secondary outcomes were the individual components of the primary outcome, and recurrence of cAFL. Adverse outcomes included hospitalization for congestive heart failure (CHF), stroke or transient ischemic attack (TIA), major or clinically relevant non-major bleeding, cardiovascular death, and overall mortality. Hospitalization for CHF was defined as at least one overnight stay in the hospital with symptoms and signs of CHF. Stroke was defined as a new focal neurological dysfunction with clinical, imaging, or pathological evidence of focal infarction due to ischemic, hemorrhagic or undetermined origin. Major bleeding included fatal bleeding, symptomatic bleeding in a critical area or organ (intracranial, intraspinal, intraocular, retroperitoneal, intra-articular, pericardial or intramuscular with compartment syndrome), and bleeding causing a fall in hemoglobin level of ≥2 g/dL within 7 days, or leading to a transfusion of ≥2 units of blood transfusion. Cardiovascular death included any death with documented cardiovascular origin, and included fatal bleedings. Standardized validation of study outcomes was performed by independent physicians.

### 2.5. Statistical Analysis

Baseline characteristics were shown as unstratified. The distribution of continuous variables was checked by visual inspection of the histogram, and evaluation of skewness and kurtosis. As all variables were normally distributed, they were presented as means (±standard deviations (SD)). Categorical variables were presented as counts (percentages). Kaplan–Meier survival curves were built to assess the incidence of AF or AT after cAFL ablation. Sensitivity analyses included stratification for sex and age (<mean age vs. ≥mean age). Cox proportional-hazards models were built to investigate potential predictors of the primary outcome. First, potential predictors were added separately in individual models. As no predictor was significantly associated with the primary outcome in individual models, we did not perform further multivariate analyses. Potential predictors were selected based on prior literature, availability, and biological plausibility; these were sex, age, BMI, history of CHF, diabetes, hypertension, coronary artery disease, left ventricular ejection fraction (LVEF), left atrial volume, prior tachycardia induced cardiomyopathy, the CHA_2_DS_2_-VASc score [8], the EHRA score, intake of anti-arrhythmic drugs (AAD), and intake of betablockers. A two-sided *p*-value < 0.05 was considered statistically significant. All statistical analyses were performed using SAS 9.4 (SAS Corporation, Cary, NC, USA).

## 3. Results

Baseline characteristics of the 309 patients undergoing cAFL ablation without prior documented AF are shown in Table 1. The mean (SD) age was 67.9 (10.5) years and 15.2% were female. The mean (SD) CHA_2_DS_2_-VASc score was 2.4 (1.5), 57 (18.5%) patients had a history of CHF, 193 (62.5%) patients had arterial hypertension and 52 (16.8%) patients were diabetic. During the ablation procedure, bi-directional conduction block across the CTI was reached in all patients. The great majority of patients (90.3%) were anticoagulated. The mean (SD) LVEF was 50.7 (13.5) % and the mean (SD) left atrial volume was 39.5 (17.8) mL. 

Of the 860 scheduled follow-up visits, 275 (31.9%) were missed. Of the 275 missed visits, 97 (35.3%) fell into the early phase of the current COVID-19 pandemic, from March 2020 to December 2020. One scheduled follow-up visit was missed by 107 (34.7%) patients missed and two visits were missed by 84 (27.3%) patients.

**Figure 2:**
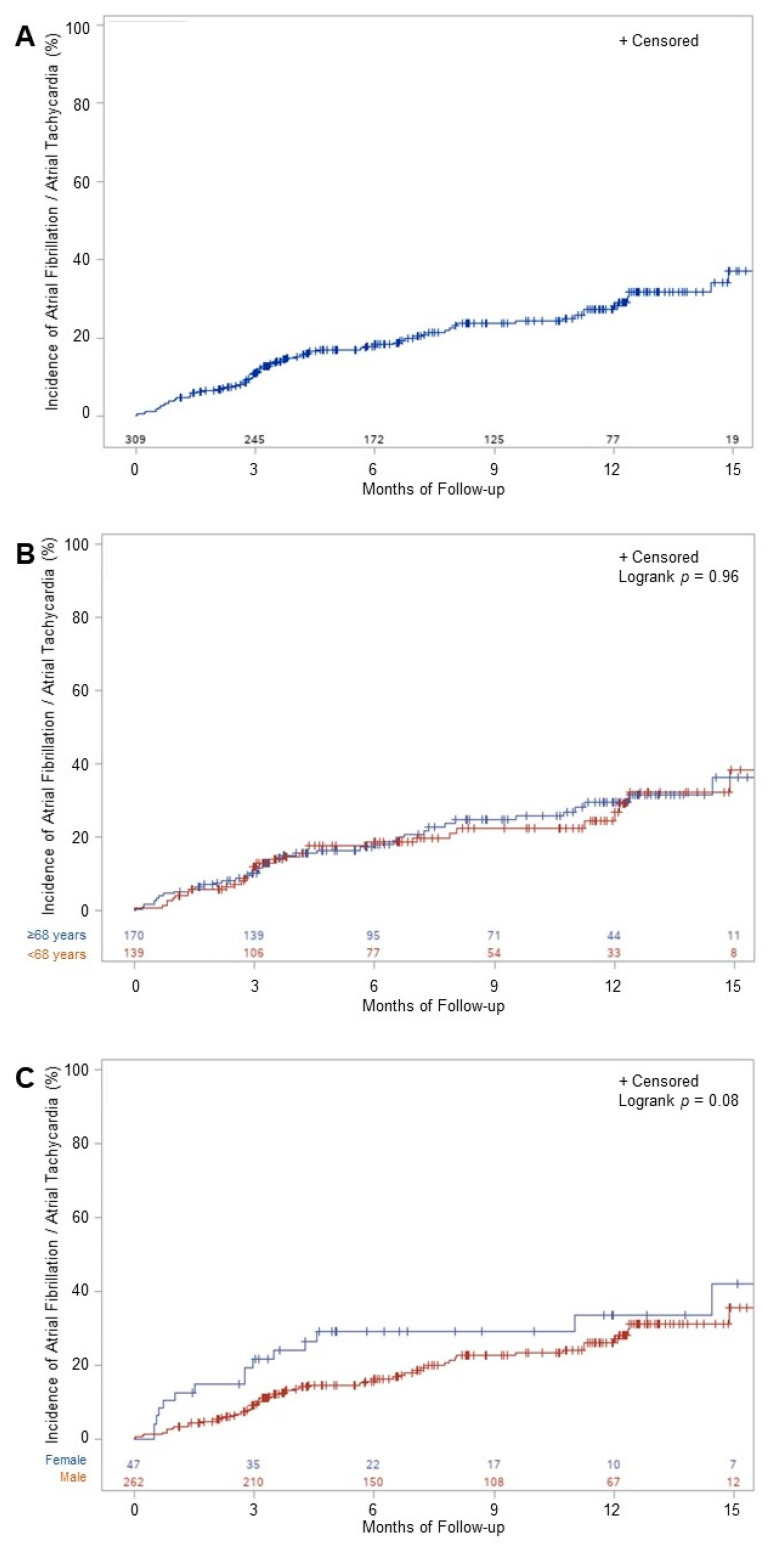
Incidence of atrial fibrillation or atrial tachycardia after ablation for CTI dependent atrial flutter overall (**A**), stratified by age (**B**) and sex (**C**). AF = atrial fibrillation.

Over a mean (SD) follow-up of 12.2 (9.2) months after the index procedure, the primary outcome occurred in 73 patients, corresponding to 11.7% at 3 months, 18.4% at 6 months and 28.2% at 12 months of follow-up (Figure 2A). AF was found in 64 patients, AT in 9 and both in 2 patients. Of the 66 patients with documented AF, the Holter-ECG identified 40 (60.6%) patients and only clinical follow-up was needed for the remaining 26 (39.4%) patients. All AT occurrences were detected by clinical follow-up. Sensitivity analyses showed no difference in the primary outcome in younger versus older patients (*p* = 0.96), or in females compared to males (*p* = 0.08) (Figure 2B,C). Of the 38 patients treated with AAD at baseline, 11 (29%) showed AF or AT during follow-up, with no difference in survival analyses for the primary endpoint, compared to the patients without AAD (logrank-*p* = 0.65). cAFL recurred in 22 (7.1%) patients. 

Adverse events during follow-up in the 73 patients with AF/AT included hospitalization for CHF in 5 (6.8%) patients, stroke in no patients, major or clinically relevant non-major bleeding in no patients, cardiovascular death in 1 (1.4%) patient and overall death in 3 (4.1%) patients. In the 236 patients without AF/AT during follow-up, 3 (1.2%) patients were hospitalized for CHF, 2 (0.8%) patients suffered a TIA, 4 (1.7%) patients had a major or clinically relevant non-major bleeding, 1 (0.4%) patient died due to a cardiovascular cause and 4 (1.7%) patients died overall. The first patient had a TIA with temporary, left-sided leg paresis during interrupted oral anticoagulation due to hysterectomy, without evidence of ischemia or bleeding from both the brain CT and MRI. The second patient had a TIA while on aspirin, with a planned change to clopidogrel without other anticoagulation. In both patients, AF was not detected at the time of the TIA, or the remaining follow-up. Overall, two (0.6%) patients withdrew study consent.

The associations of potential predictors with the primary outcome are shown in Table 2. Except for coronary artery disease (hazard ratio [95% confidence intervals] 1.03 [1.01; 1.05], *p* = 0.01), no variable was significantly associated with incidents of AF or AT.

## 4. Discussion

Atrial fibrillation, or AT, was detected in 28% of patients undergoing CTI dependent atrial flutter ablation, and with no prior evidence for atrial fibrillation, using a standardized follow-up over 1 year. Coronary artery disease was the only predictor for AF or AT occurrence after CTI ablation. We did not find a difference in detected AF between male and female, or younger and older patients.

The rate of detected AF after cAFL ablation strongly depends on the intensity of monitoring, and on the duration of follow-up [1]. In a recent meta-analysis, a purely symptom- driven monitoring strategy detected AF in approximately 12% of patients undergoing cAFL ablation without prior documented AF. This rate increased to about 46% with intensive monitoring, which included long-term Holter monitoring over more than 7 days or implantable loop recorders [1]. A routine monitoring strategy, with less than 7 days of Holter monitoring per year, showed an AF detection rate of 19% [1], which is outperformed by our current detection rate of 28%. The detection rate can be further increased by continuous rhythm monitoring using implantable loop recorders, as shown by Mittal et al. in a cohort of 20 patients, with AF detected in 55% [9]. However, the clinical significance of short AF episodes, that may be picked up by continuous monitoring, is not yet known. Currently, no clear guideline recommendations exist on the optimal follow-up approach [6]. Our standardized follow-up and monitoring approach followed the recommended follow-up after PVI [6]. It showed a high rate of detected AF, was clinically feasible and had a low withdrawal rate, below one percent. We observed a TIA rate of 0.6%, which is lower than the stroke rate reported in the major randomized trials comparing direct oral anticoagulants (1.2–1.7% per year), and warfarin (1.5–2.0% per year) [10,11,12]. However, in the patient on anti-platelets during the TIA, a more intensive follow-up might have detected AF with the initiation of oral anticoagulation before the event, and may have prevented it.

Ideally, patients with a higher baseline risk for developing newly diagnosed AF after cAFL ablation should be identified by risk factors, in order to direct resources for a more intense follow-up of these patients. Although some prior studies have identified such risk factors, including age [13], left ventricular dysfunction [1,14,15], left atrial dilatation [2,16] and arterial hypertension [1], these were not consistent across studies [17]. In our study, we only identified coronary artery disease as a significant predictor for recurrent AF or AT. However, this single and common risk factor does not allow researchers to reliably risk-stratify patients for monitoring. While it is conceivable that more specific risk predictors for AF (for example, the burden of atrial ectopic premature beats on Holter monitoring [18]) might be more consistent across different patient populations, these have to be tested in prospective studies. Until such data are available, a standardized approach for all patients should be preferred. Ultimately, the clinical utility of monitoring for AF depends on the therapeutic consequences. Current guidelines recommend anticoagulation in cAFL patients who are similar to AF patients, but independent of the presence of AF [19]. However, most data for the thromboembolic risk of cAFL patients were derived from cohorts that also included AF patients [20]. The thromboembolic risk in patients with only cAFL seems to be lower than that in patients with additional symptoms, or only AF [21]. Moreover, to our knowledge, no prospective, randomized evaluation of the CHA_2_DS_2_-VASc score exists for anticoagulation use in patients with only cAFL. Finally, observational data from registries show that, despite guideline recommendations, anticoagulation is frequently stopped in a large proportion of cAFL patients after ablation in clinical practice [21,22]. Therefore, we believe that it is still worthwhile to screen for AF, with regard to anticoagulation. Besides anticoagulation, detection of AF is also relevant for patients with a reduced left ventricular ejection fraction who might benefit from AF catheter ablation [6,23].

The close interrelationship of AF and cAFL is most likely due to shared risk factors, and common arrhythmia mechanisms influencing each other [3]. In the majority of cAFL, AF, or frequent atrial premature beats leads to a functional line of block between the superior and inferior vena cava, a critical component for cAFL [4,5]. Thus, AF seems to be a necessary prerequisite for cAFL initiation, except in instances where an anatomical, fixed line of block is present. The theory of AF being necessary to induce cAFL led to several studies investigating the incremental value of adding PVI to CTI ablation, in patients without prior documented AF. All three studies showed a reduction in AF occurrence during follow-up, compared to patients without additional PVI [24,25,26]. To further investigate the value of PVI in cAFL patients without prior documented AF, the ongoing ‘Cryoballoon Ablation as First Line Treatment of Atrial Flutter (CRAFT)’ study (NCT03401099) randomizes patients to either CTI ablation or to PVI alone [27].

The strengths of the current study include the large numbers of unselected, well-characterized patients with a standardized follow-up. Several limitations have to be taken into account when interpreting the results. Firstly, the observational nature of our study does not allow us to establish causality. Secondly, the follow-up was limited to one year; therefore, we might have missed late occurrences of AF. Additionally, we may have missed asymptomatic episodes of AF, due to the non-continuous AF monitoring. However, both the duration and intensity of follow-up were intentionally chosen, to maximize clinical utility and patient acceptance. Thirdly, no control group with only clinical or more intensive follow-up was available. Fourthly, we did not assess the cost-effectiveness of the follow-up approach. Fifthly, we did not investigate specific Holter-ECG parameters, such as atrial premature ectopic beats available, as potential predictors for the primary outcome.

In conclusion, AF was detected in a large number of patients undergoing CTI dependent cAFL ablation, using a standardized follow-up with intermittent ECG monitoring. Coronary artery disease was the only predictor for AF or AT occurrence after CTI ablation. Our results support a standardized follow-up in all patients after CTI ablation without prior documented AF, and may have important clinical implications with regards to the anticoagulation management.

## Figures and Tables

**Table 1:** Baseline characteristics.

	Overall *n* = 309
Sex (female), *n*	47 (15.2)
Age, years	67.9 (10.5)
BMI, kg/m^2^	27.7 (5.3)
CHA_2_DS_2_-VASc score, points	2.4 (1.5)
EHRA score, points	2.2 (0.7)
Prior heart failure, *n*	57 (18.5)
Tachycardia-induced cardiomyopathy, *n*	22 (7.1)
Hypertension, *n*	193 (62.5)
Diabetes, *n*	52 (16.8)
Coronary heart disease, *n*	52 (16.8)
LVEF, %	50.7 (13.5)
Left atrial volume, ml	39.5 (17.8)
Treatment, *n*	
Oral anticoagulation, *n*	279 (90.3)
AAD	38 (12.3)
Beta-blocker	196 (63.4)
RAAS-inhibitor	114 (36.9)

Continuous variables are shown as means (standard deviation). Categorial variables are shown as counts (percentages); AAD = anti-arrhythmic drug; BMI = body mass index; LVEF = left ventricular ejection fraction; RAAS = Renin–Angiotensin–Aldosterone system. CHA_2_DS_2_-VASc: Congestive heart failure, Hypertension, Age, Diabetes, Stroke, Vascular disease, Sex category; EHRA: European Heart Rhythm Association.

**Table 2:** Predictors for incident atrial fibrillation or AT after ablation for CTI dependent right atrial flutter.

Predictor	Univariate	
	HR (95% CI)	*p*-Value
Sex, female	1.63 (0.93; 2.84)	0.09
Age, per 1 year	1.00 (0.98; 1.02)	0.98
BMI, per unit	0.98 (0.93; 1.03)	0.17
CHA_2_DS_2_-VASc, per point	1.03 (0.88; 1.20)	0.72
EHRA score, per point	0.77 (0.55; 1.07)	0.11
Prior heart failure	0.94 (0.52; 1.72)	0.85
Diabetes	0.99 (0.54; 1.80)	0.97
Hypertension	0.79 (0.50; 1.26)	0.33
Coronary heart disease	1.03 (1.01; 1.05)	0.01
Tachycardia induced cardiomyopathy	0.77 (0.32; 1.82)	0.54
LVEF, per 1%	1.00 (0.98; 1.01)	0.66
LA volume, per 1 mL	1.01 (0.99; 1.03)	0.25
AAD	0.98 (0.84; 1.14)	0.77
Beta-blockers	1.18 (0.72; 1.92)	0.51

AAD = anti-arrhythmic drug; BMI = body mass index; LA = left atrium; LVEF = left ventricular ejection fraction. HR: Hazard ratio; CI: Confidence interval; CHA_2_DS_2_-VASc: Congestive heart failure, Hypertension, Age, Diabetes, Stroke, Vascular disease, Sex category.

## Data Availability

The data supporting this study is available from the corresponding author upon reasonable request.
